# Understanding temperature-rain data using ID3 based concept reduction technique in FCA

**DOI:** 10.1038/s41598-025-02652-1

**Published:** 2025-05-25

**Authors:** S. Usharani, S. Kaspar

**Affiliations:** Department of Mathematics, Vellore Institute of Technology, Vellore, Tamil Nadu 632014 India

**Keywords:** Concept, Concept lattice, Entropy, Formal concept analysis, ID3 algorithm, Information gain, Climate sciences, Mathematics and computing

## Abstract

Proper understanding of rain yield along with the relevance factors and their extent of relation in the yield of rain is very important to maintain a smooth life style in every one’s life. The definitive classification of mean temperature and heaviest rainy days depends on the weather changes that occur seasonwise during any year. In reality, visualizing the effects of climatic changes such as temperature in the rain-yield during over a period of years is very difficult and there is no method or tool to help us in this aspect. Formal concept analysis (FCA) which is a mathematical model that expresses the relationship between various features and entities in terms of pairs called concepts. These concepts are hierarchically related to form a unique concept lattice which is a diagrammatical view of the information available. In this paper, an approach to facilitate the understanding of temperature-rain data of Vellore district with the use of data collected for the recent 15 years period is presented. For the analysis, the mean temperature data is preprocessed seasonwise over the years. In a similar manner the rainy days also preprocessed seasonwise over the years. We illustrate the process of extracting meaningful information from the data with the use of FCA. In this process an ID3 algorithm based method is employed to identify more important features from the context. These important features are used to compress the concepts obtained from FCA and thereby reduce meaningful information. The efficiency of the proposed method is validated using few efficient metrics available in literature.

## Introduction

In early 1980s, Rudolf Wille developed a mathematical theory called formal concept analysis (FCA). Over the past two decades, FCA has been used in numerous fields such as knowledge discovery, information retrieval and software engineering. It is focused on conceptual thinking and the formalization of concepts. It describes the mathematical structure of available information^1,2^. FCA is a mathematical theory which embodies the conceptual understanding of concepts and their relationships. FCA is a mathematical theory that represents a conceptual knowledge of ideas and their hierarchies. It formally establishes the relationship between an idea’s extension and intention, where extension and intension respectively refer to set of objects and attributes that are commonly related. An axiom states that growing intent entails decreasing extent and vice versa. It is possible to derive a concept hierarchy from a given data set by using lattice theory as a basis. Thus, FCA is a complement to the standard ontology engineering method, which involves modeling the concept hierarchy by hand. Whereas FCA considers both the extensional and intensional aspects of concepts to be equally important. As a result, FCA enhances other conceptual knowledge representations, and combining it with other representations has been the subject of numerous articles and they have presented a different strategy on the application in FCA. FCA is origin and growth is detailed in^3^. FCA has huge real life applications to understanding the information present in many contexts. For the benefit of readers we list some of them below.

A virtual learning environment (VLE) or learning management system (LMS) refers to the set of software tools that facilitates the conduct of courses, meetings, training, and other activities through online mode. Sumangali et al.^4^ constructed the formal context of an open-source LMS for an e-learning environment and assumed object as LMS tools and attributes as the features provided by them. In 1920, Jan Lukasiewicz introduced many-valued logic, and Han and Kamber in 2006 used it in many-valued contexts of FCA and databases. Bernhard Ganter et al.^5^ proposed a heuristic algorithm based on scaled many-valued context. The best open bibliographic database, search engine, and knowledge graph for computer science papers is the DBLP (Digital Bibliography and Library Project) computer science bibliography. Salesh Alwahai et al.^6^ derived and classified the journal of DBLP datasets and analyzed the relation between journals and topics. Ley^7^ provided the DBLP-based classification. Krohn et al.^8^ presented the acquisition of new knowledge and enhanced the interactions between knowledge workers. In next section discuss the motivation lying behind this work.

### Motivation

#### FCA framework

Belohalvek et al.^9^ presented formal concepts with large values that are considered more important and selected the ’important concepts’ that rejected the small number of concepts. The study of solving problems with significant features at different and/or scales of space is known as multi-scale mathematics. A technique for determining the best scale combination for every characteristic in a multi-scale decision table (MDT) is called optimal scale selection. Dongxiao Chen et al.^10^ established the idea of a multi-scale formal framework, and when the scale varied, the objects that belong to each attribute undergo monotonous changes. They have discussed optimal scale selection and multi-scaled decision context in FCA . Radek Janostik et al.^11^ developed two methods for building blocks and an algorithm for computing spanning patterns in logical analysis with the use of FCA. Yoshikia Sami et al.^12^ determined the performance of data for practice-based office E-learning in FCA. They further compared the implication relation between the true/false questions and analysed it using statistical analysis methods. Anna Formica^13^ evaluated concept similarities between Interodinal scaling FCA (IFCA) and ordinal scaling FCA (OFCA). Sean Tull^14^studied two corresponding tensors of sub-lattices, the category of bonds between contexts, and the explanation in the lattice tensor. Amit Kumar Mishra et al.^15^ suggested weights under terrorist network mining to find the node that had an impact on other terrorists. They concentrated on the information about the terrorist network using concepts & their hierarchies which is accessible worldwide. Tong-Jun Li et al.^16^ thoroughly explored a partial order relation among sub contexts is in order to introduce the decision rules and dependencies between the formal context attributes. We discuss about Iterative Dichotomiser 3 (ID3) algorithm and its use in various areas in recent years. The decision tree algorithm, ID3 algorithm, handles only categorical attributes.

#### ID3 algorithm

The extent of information lying in any data means can be measured using information gain which uses another information entropy in the computation process. Information entropy corresponds to the extent of uncertainty present in an information system. It’s a probabilistic value ranging from. It is faster and uses the entire data set. A full description of ID3 algorithm appears in^17^ for the first time. Many author employed ID3 algorithm to identify the quality of information present in the data sets. For example Fadwa Aaboub et al.^18^ used the ID3 algorithm decision tree technique to employ the information gain metric to evaluate the significance of the attribute. Hong Fang Zhou et al.^19^ used five types of decision algorithms with ID3 and proved that the constructed decision trees are basically equal. Patsita Wetchaoram et al.^20^ proposed the feature selection and decision tree construction based on a hierarchical concept model using FCA. They select the top three high levels of hierarchical structure in both models. Both models perform equally, with no difference in the classification . ID3 algorithms can be found in serveral articles such as^21–25^.

In this article, the authors have proposed a method to extract meaningful information by reducing large contexts into smaller, more manageable ones. To achieve this, the ID3 algorithm is applied to prioritize the attributes within the context. Finally, the experimental results are validated through comparison measures.

#### Climate change

The weather condition and the rain yield, influence the life style of people in any country. In India, the weather conditions and amount of rain yield are closely observed by the Indian meteorological society which is located at Pune. It observes four seasons with certain local variations: Winter, Summer, Monsoon and Autumn. The majority of Indian climate can be considered tropical in nature, even though the Tropic of Cancer, the line between the tropics and subtropics, crosses through the center of the nation. Monsoonal and other weather patterns may be highly unpredictable in India, as they are in much of the tropics. Periodic natural calamities such as heat waves, floods, cyclones, and epochal droughts have caused millions of people to be displaced or killed. Due to climate change efforts brought on by humans, these types of climate catastrophes are probably going to occur less frequently and with less intensity.

Additional effects of global warming include rising sea levels, inundation of low-lying coastal areas of India, and ongoing and future changes in the vegetation. Summer season brings with it extremely hot temperatures. Most of the rainfall occurs between May and November, and sustains lush wood lands, swampy areas, and other plants during the largely dry year. The driest months are December through March, when rainy days are infrequent. Parts of these locations have exceptionally rich tropical wet forest biodiversity because of the strong monsoon rains. It is more typical to have a tropical, rainy and dry climate in India. With the exception of a semi-arid rain shadow, it covers the majority of interior peninsular India and is noticeably drier than regions experiencing a tropical monsoon type of climate. The winter and early summer seasons are long and dry, with average temperatures above 18 °C (64 °F). In May, temperatures in low-lying areas can reach over 50 °C (122 °F), causing heat waves that have the potential to harm hundreds of Indians at once. Summers are especially scorching. During the rainy season, which lasts from June to September, the region receives an average of 750 to 1,500 mm (30 to 59 in) of rainfall annually. When the dry northeast monsoon starts in September, Tamil Nadu (State) and Pondicherry (Union Territory and neighbor to Tamil Nadu) from India receive the majority of India’s precipitation, leaving the other states largely dry. Tamil Nadu state has a tropical climate with year-round high temperatures, with the exception of the monsoon season. Since Chennai (Madras) the capital of Tamil Nadu is located on the thermal equator, there is little difference in temperature. Because of its significance and location in relation to Chennai, Vellore also possesses similar climate and often known as the Second Madras.

Climate change is one of the important factors in human life. When examining geographical phenomena, the term ’regional scales’ refers to a broad area, such as a state, province, or region. Maria Chanra Karpidou et al.^26^ used a multi-model strategy that included a mechanistic model and an ensemble of other correlation niche models to assess the influence of climate change on the risk of being affected by malaria. It can assist in the process of well-informed decision-making at the national and regional levels . Ellen et al.^27^ assessed the effects of climate change an human health issues due to ozone. Olutayo Odunola et al.^28^ examined the effects of climate change-related diseases on children’s health. They have recommended the government agencies to improve the quality of health services rendered to children.

The route scenarios which assist nations in meeting their climate and sustainable development objectives are models that investigate economic and emissions scenarios. One mathematical tool for simulating real-world issues is the Caputo fractional derivative. Anwarud Din et al.^29^ investigated the climate change model using Caputo fractional derivative and got the approximate solution, using the iterative numerical technique of the problems . Jung Eun Kin et al.^30^ used climate change data based on representation pathway scenarios to analyze how malaria is spread by climate changes . The mathematical functions known as intensity duration frequency curves illustrate the connection between an event’s intensity, duration, and frequency. Pao Shan Yu et al.^31^ created a regional intensity duration frequency based on the annual maximum rainfall data. Using intensity duration frequency scaling formulas, they determined the piecewise simple scaling hypothesis with a Gumbel distribution in . Moctor Dembele et al.^32^ evaluated and compared the satellite-based rainfall products in West Africa. They used continuous statistics to estimate and reproduce rainfall amounts and categorical statistics to evaluate rain detection capabilities. Wissanupon Kliengchuacy et al.^33^ investigated temperature, humidity, and precipitation and used the Mann-Kendall test and innovative trend analysis to predict the future situations.

From the literature, we notice that to our knowledge the use of FCA to analyze weather conditions is not available so far. So this is the first article which analyzes temperature-rain data with the use of FCA.

In view of importance of climate change behaviours, to understand and predict any climatical causes such as rain, it is very important to analyses the patterns and structures resulting out of temperature and rain fall seasonally and annually. One such attempt is made in this article with the use of FCA tool and ID3 algorithm. In this article, some background information on FCA, concept lattice and weather (temperature and rain) are provided. In “Basic definitions with example Definition: FCA” section, basic definitions and notions of FCA and concept lattice are provided and the same has been illustrated with the use of a real life context. “Data analysis” section describes how FCA is used to create a visual overview of the fifteen years temperature-rain data in Vellore district (January 2008–December 2022). Classification of seasons on weather (temperature) and rain are constructed seasonwise, yearwise as generally followed and accepted by society. In “Iterative Dichotomiser 3 (ID3) algorithm—an overview” section, definitions of ID3 algorithm and steps involved in it are discussed. In “Experiment with ID3 algorithm” section, ID3 algorithm is applied to determine the important concepts from which concepts are extracted using the relative importance. In “Experimental results” section, the method is further validated using available metrics. The article is concluded in “Conclusions” section.

## Basic definitions with example

### Definition: FCA

Wille^1^ and Ganter and Wille^2^ A formal context (*U*, *V*, *R*) consists of two sets U and V and a relation R between U and V. The elements present in U and V are known respectively as objects and attributes of the context. We say that u and v are related and write *uRv* or $(u, v) \in R$, if the object u has the attribute v. For any formal context (*U*, *V*, *R*), a pair of dual operators $X \subseteq U$ and $A \subseteq V$ are defined by:

$$X^*$$=$$\{v \in V | uRv,\forall u \in X\}$$ and

$$A^*$$= $$\{u \in U | uRv,\forall v \in A\}$$.

If $X^*= A$ and $A^*= X$, the pair (*X*, *A*) is said to form a formal concept. In this case, X and A are given the tags extent and intent of the format concept (*X*, *A*).

### Definition: Conceptual hierarchy

Let the set of all concepts be denoted by *B*(*U*, *V*, *R*). For any two concepts $(X_{1}, A_{1})$, $(X_{2}, A_{2})\in B(U, V, R)$, the subconcept and superconcept relation denoted by ‘≤’ is defined as follows:

$(X_{1}, A_{1}) \le (X_{2}, A_{2})\Leftrightarrow X_{1}\subseteq X_{2}(\Leftrightarrow A_{1}\supseteq A_{2})$. The lower and upper concepts are called respectively as subconcepts and superconcepts.

### Definition: Concept lattice

A concept lattice, represented as *L*(*U*, *V*, *R*), is the complete lattice formed by the family of all formal concepts (*U*, *V*, *R*) using the hierarchy of concepts.

The notions of contexts, extent, intent, concepts and the construction of concept lattices are discussed explanatorily in the following books^34–36^.

### Example

The Table 1 presented here describes few types/sources of water and their PH concentration levels classified categorically (net sources).

**Table 1:** A formal context (*U*, *V*, *R*).

Source of water (PH Levels)					
Types of water	a(4-5)	b(5-6)	c(6-7)	d(7-8)	d(8-9)
TW				X	
BW			X	X	
OW					X
SW	X	X			
IW			X	X	
MW		X	X	X	X
DW		X	X		

**Fig. 1:**
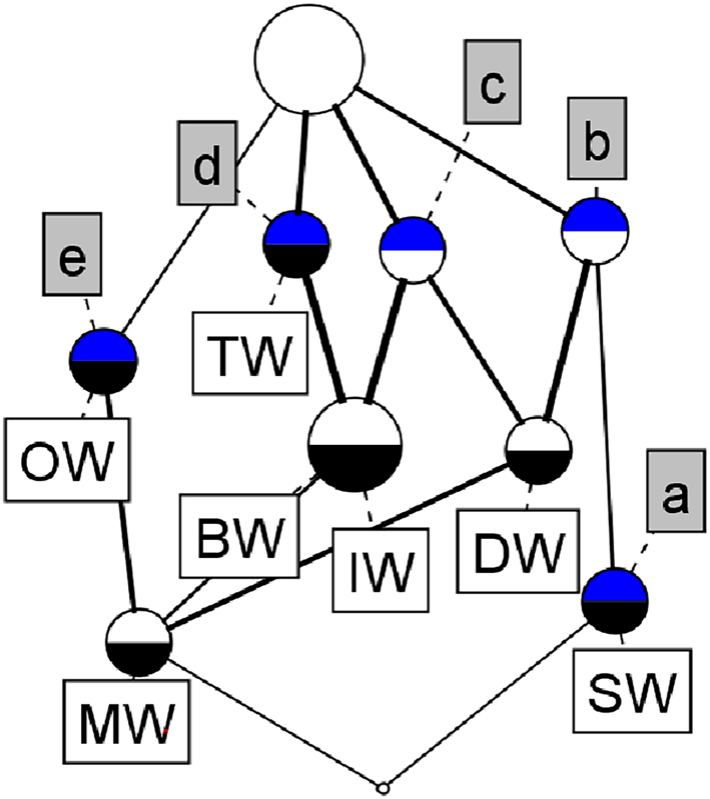
Concept lattice derived from the formal context Table 1.

Table 1 is a formal context and Fig. 1 represents its concept lattice (concept count is 10, edge count is 14, lattice height is 4, and width estimation is (3,6)). In Table 1 the value present (X) in any cell implies the existence of the relationship between the corresponding row object and corresponding column attribute; the absence of a relationship between objects and attributes are denoted by empty cells. The abbreviations used for the objects are: Tap Water (TW), Bottled Water (BW), Ocean Water (OW), Sparkling Water(SW), Ice Water (IW), Mineral Water (MW) and Distilled Water (DW). The columns correspond to various PH values found in the substances. The bottom most element represented by the concept lattice in Fig. 1 contains empty extent and its intent possesses all the attributes of the context. Similarly, the topmost element of the lattice contains an extent that has the entire objects of the context whereas, its intent is empty. In any concept lattice, objects increase as we trace from bottom concept to top concepts upwards while attributes increase from top concept to bottom as we trace downwards. Upper concepts of the lattice form the super-concepts whereas the lower ones form the sub-concepts by subset relation. In any concept lattice, every concept contains the set of objects that lie in concepts beneath it, which are joined by lines downwards apart from those objects that are introduced at that concept. Similarly, we can say that every concept contains the set of attributes of every concept aloft which are joined by upward lines apart from those attributes attached to it first time.

The context in Table 1 contains 10 concepts which form a concept lattice as shown in Table 1. Each node in the concept lattice corresponds to a concept. The extent of a concept (or node) is the set of all objects which are added to the concepts connected by the descending path from that concept. Similarly, the intent of a concept is the set of all attributes which are added to the concepts (nodes) that are connected by the ascending path from that concept. Objects For example, the node labeled with MW represents a concept whose extent and intent pair is ({MW}, {b, c, d, e}). In this manner, we can understand the remaining concepts.

FCA has wide variety of applications in the study or analysis of data in various domains such as feature selection, decision support systems, text and document analysis, data visualization, data cleaning and preprocessing, knowledge discovery and representation, clustering and classification, association rule mining, semantic analysis and ontology engineering, data analysis, and software engineering, etc.

In the following section, we experiment the weather data to extract the meaningful information using FCA.

## Data analysis

Weather data collected from Indian Meteorological Department, National Data Center (NDC), Pune (The Data Supply Portal http://dsp.imdpune.gov.in/) is presented in Table 5. The information presented in Table 5 is an abstract of the data collected from the Data Supply Portal (DSP) Pune, India.

We have collected fifteen years temperature-rain data pertaining to Vellore district monthwise between January 2008 and December 2022.The temperature levels over any period vary over some range. In order to incorporate the variations effectively the mean temperature data has been collected monthwise from DSP. The climate differs vastly seasonwise. Accordingly, the temperature and rainfall also fluctuate massively across the seasons. So as a step of preprocessing, the collected data has been classified into seasonwise data in order to reduce the size complexity of the data. For this purpose, we have considered the four seasons of India as decided by the India Meteorological Department (IMD) comprising the months as follows:

Winter—December, January and February

Summer—March, April and May

Monsoon—June, July, August and September and

Autumn—October and November

The temperature levels over the years vary between 27–42 °C in Vellore, a city from the state of Tamil Nadu, India. The temperature levels are categorized seasonwise as low, medium and high and are abbreviated. For example WL refers to the presence of ‘low temperature during winter season’. Similarly other abbreviations can be understood as listed below in Table 2:

The categorization of temperature levels are due to arbitrary threshold ranges as Low: 27.1–32.1, Medium: 32.2–37.2, and High: 37.3–42.3.

**Table 2:** Abbreviation of seasons.

Temperature levels				
Seasons	Low	Medium	High	Rain
Winter	WL	WM	WH	WR
Summer	SL	SM	SH	SR
Monsoon	ML	MM	MH	MR
Autumn	AL	AM	AH	AR

The rain levels are split into less rain, rain seasonwise as listed in the Table 3. Further the same is also considered yearwise, aggregating the four seasons. Further, yearwise rain yield is split into three categories as Low, Medium, High as in Table 4.

**Table 3:** Classification of rain seasonwise.

Seasons	Level 1 (Low rain)	Level 2 (Rain)
Winter	0–9 days	10 days and above
Summer	0–9 days	10 days and above
Monsoon	0–27 days	28 days and above
Autumn	0–16 days	17 days and above

Apart from seasonwise categorization of rainy days, the decision attribute viz., the annual Total Rainy (TR) Days data is a categorical data and it is dealt accordingly to handle it as a formal context. The classifications on TR are done by splitting the range of yearly rainy days over the entire period into three equal intervals as TR1, TR2 and TR3 as shown in Table 4. Such a classification on the decision about the rainfall over a year is necessary since we cannot easily conclude annual rainfall in any region with a mere binary attribute yes/no. In this regard, the classification on the decision attribute rain is made using the information presented in Table 3. After the formalization of the available information on weather data the information system can be effectively depicted by means of a formal context.

**Table 4:** Classification of total rain level.

Rain level	TR1-Low rain	TR2-medium rain	TR3-High Rain
No. of days	Upto 53 days	54–65 days	Above 65 days

**Table 5:** Context of temperature-rain days in Vellore District.

	WL	WM	WH	WR	SL	SM	SH	SR	ML	MM	MH	MR	AL	AM	AH	AR	Decision Attribute
2008	X	X				X	X	X		X			X			X	Medium
2009	X	X				X	X			X	X		X	X			Low
2010	X	X		X			X	X		X	X	X	X	X		X	High
2011	X	X				X	X			X	X		X	X		X	Medium
2012	X	X					X			X	X	X	X	X		X	Medium
2013	X	X				X	X			X		X	X	X			Low
2014	X	X				X	X			X	X		X				Low
2015	X	X				X				X			X	X		X	Medium
2016	X	X				X	X			X		X		X			Low
2017	X	X				X	X			X	X	X	X			X	Medium
2018	X					X	X			X			X			X	Low
2019	X					X	X			X	X	X	X	X			Medium
2020	X			X		X	X			X	X	X		X		X	High
2021	X					X	X			X		X	X			X	High
2022	X	X				X	X			X		X	X			X	Medium

**Fig. 2:**
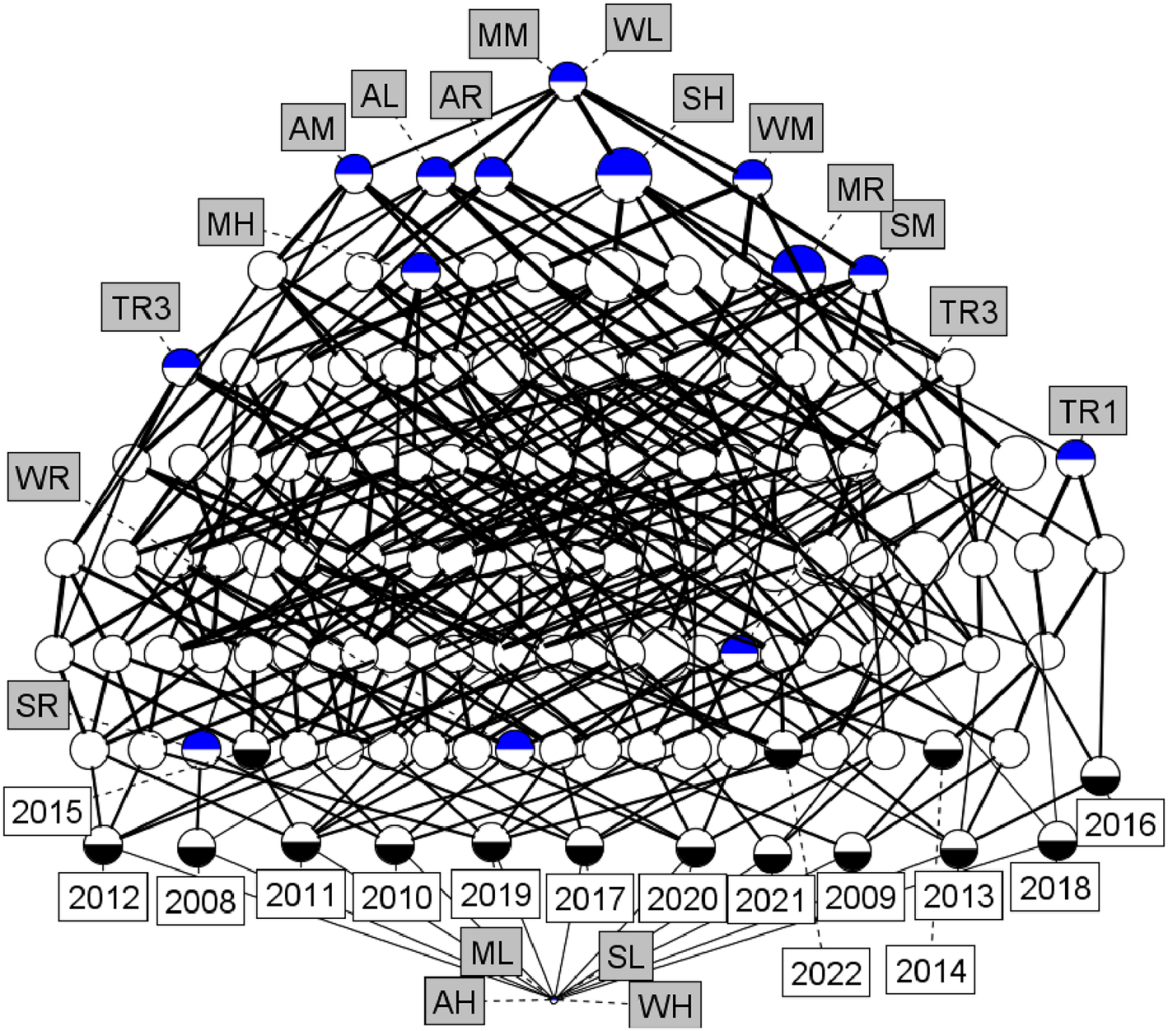
Concept lattice derived from the formal context Table 5.

Using the notions and terms defined earlier on concepts and concept lattices, we get a huge concept lattice form Table 5 as shown below in Fig. 2, which is very complex and difficult to understand the required information. This original concept lattice has totally 145 numbers of concepts with edge count 424. The height of the lattice is 9 and its width estimation is (28,136). So obviously complexity is very high and we cannot understand anything from the concept lattice.

In all concept lattices, the concept corresponding to the bottom node consists of an extent with the least number of objects and the entire set of attributes as intent. Similarly, at the top node the concept has an entire set of attributes as extent and the least number of attributes as intent. For example, the concept lattice in Fig. 2 has the concept ({15 objects},{WL, MM}), at the top node and the concept at the bottom node is ( ,{19 attributes}). The remaining concepts can be understood as explained earlier in “2.4” section.

Next, we discuss the Duquenne-Guigues base of implications. In a concept lattice, an implication is a relationship between two sets of attributes, A and B, that expresses that every object with each attribute in A also has each attribute in B. This relationship is written as $A\Rightarrow B$.

There are 37 implications for the weather context presented above in Table ??. We list them below. The notions are presented in the following order: Serial No<Number of objects>Premise $\Rightarrow$ Conclusion. An empty premise is given by “ ”, which means that this implication has an empty premise and holds for all objects from context.

1 $$< 15>$$ $$\Rightarrow$$ WL MM;

2 $< 2>$ WL SR MM $\Rightarrow$ WM MM SM AL AR;

3 $< 10>$ WL WM SM MM $\Rightarrow$ AL;

4 $< 8>$ WL MM MH $\Rightarrow$ SM;

5 $< 5>$ WL SM MM MH MR $\Rightarrow$ MM;

6 $<13>$ WL MM AL $\Rightarrow$ SM;

7 $< 4>$ WL WM MM SM MM MR AL $\Rightarrow$ AR;

8 $< 4>$ WL MM SM MM MR AM $\Rightarrow$ MH;

9 $< 10>$ WL MM AR $\Rightarrow$ SM;

10 $< 4>$ WL MM SM MM MH AR $\Rightarrow$ MR;

11 $< 6>$ WL SM MM MR AR $\Rightarrow$ MM;

12 $< 4>$ WL SM MM MH AL AR $\Rightarrow$ WM;

13 $< 4>$ WL SM MM AL AM AR $\Rightarrow$ WM;

14 $< 4>$ WL SM MM TR1 $\Rightarrow$ AL;

15 $< 2>$ WL MM MR TR1 $\Rightarrow$ WM AM;

16 $< 2>$ WL SM MM MH AL TR1 $\Rightarrow$ WM MM;

17 $< 2>$ WL WM MM SM MM AL TR1 $\Rightarrow$ MH;

18 $< 3>$ WL MM AM TR1 $\Rightarrow$ WM;

19 $< 1>$ WL SM MM AL AR TR1 $\Rightarrow$ MM;

20 $< 7>$ WL MM TR2 $\Rightarrow$ SM AL;

21 $< 3>$ WL MM SM MM MH AL TR2 $\Rightarrow$ MR;

22 $< 4>$ WL SM MM MR AL TR2 $\Rightarrow$ MM;

23 $< 6>$ WL WM SM MM AL TR2 $\Rightarrow$ AR;

24 $< 6>$ WL SM MM AL AR TR2 $\Rightarrow$ WM;

25 $< 3>$ WL MM TR3 $\Rightarrow$ MM SM MR AR;

26 $< 2>$ WL MM SM MM MH MR AR TR3 $\Rightarrow$ WR AM;

27 $< 1>$ WL WM MM SM MM MR AL AR TR3 $\Rightarrow$ WR SR MH AM;

28 $< 0>$ WL ML MM $\Rightarrow$ WM WH WR SL MM SM SR MH MR AL AM AH AR TR1 TR2 TR3;

29 $<0>$ WL SL MM $\Rightarrow$ WM WH WR MM SM SR ML MH MR AL AM AH AR TR1 TR2 TR3;

30 $< 2>$ WL WR MM $\Rightarrow$ MM SM MH MR AM AR TR3;

31 $< 0>$ WL WH MM $\Rightarrow$ WM WR SL MM SM SR ML MH MR AL AM AH AR TR1 TR2 TR3;

32 $< 0>$ WL MM AH $\Rightarrow$ WM WH WR SL MM SM SR ML MH MR AL AM AR TR1 TR2 TR3;

33 $< 1>$ WL WM MM SM SR MM AL AM AR $\Rightarrow$ WR MH MR TR3;

34 $< 1>$ WL WM MM SM SR MM MR AL AR $\Rightarrow$ WR MH AM TR3;

35 $< 0>$ WL WM MM SM MM MH MR AL AM AR TR1 $\Rightarrow$ WH WR SL SR ML AH TR2 TR3;

36 $< 0>$ WL SM MM AL TR1 TR2 $\Rightarrow$ WM WH WR SL MM SR ML MH MR AM AH AR TR3;

37 $< 0>$ WL WM WR MM SM SR MM MH MR AL AM AR TR2 TR3 $\Rightarrow$ WH SL ML AH TR1;

Here we recommend the articles^1–3^ and^34–36^ to understand the notions of intent and extent, concepts and concept hierarchies and the method of construction of concept lattices.

Information entropy is a widely used measure to measure the degree of meaningful information present in a context or data with respect to each feature/attribute. FCA focuses on the understanding of relevant information present in the dataset whereas information entropy and Information entropy determines the degree of uncertainty from which the degree of meaningful information is found by another measure called information gain. So the implementation of information entropy and information gain in the field of FCA can assist in the process of understanding more important attributes present in the context. This method can yield better results while extracting the information from the dataset. In this view, it is required to study about the information entropy. The popular algorithm which determines information entropy and information gain is the ID3 algorithm. The next section discusses about ID3 algorithm and we try to apply it to reduce the concepts produced from the formal context of temperature-rain data.

## Iterative Dichotomiser 3 (ID3) algorithm-an overview

One of the popular decision tree algorithms in machine learning is the ID3 algorithm. Ross Quinlan^17^ introduced the ID3 algorithm in 1986. It’s a greedy algorithm which divides the data set recursively into smaller and smaller subsets until every data point in each subset is a member of some class, thus creating a decision tree. It uses a top-down approach method, dividing the dataset according to information gain by recursively choosing features. During the tree-building phase, the ID3 algorithm makes decisions based on information theory metrics, including entropy and information gain.

### Definition: Information entropy

The measure of uncertainty in a random variable is called entropy. Shannon^21^ introduced this measure in 1948 and it represents the average degree of uncertainty present in the random variable, which can be regarded as a portion of the information’s content. The entropy of a dataset S is denoted by H(S) and is given by the formula:

1 $$\begin{aligned} H(S)=-\sum _{i=1}^{\ m}(P_{i}log_{m}(P_{i})), \end{aligned}$$

Where $P_{i}$ is the probability of $i^{th}$ decision in the dataset S, the summation varies over the number of decisions in the dataset. The choice of base m for the logarithm in the Shannon entropy formula given in equation (1) depends on the number of branches emanating from each node of the decision tree. Since there are 3 decisions in our context, we choose the base m as 3. This convention has already been used in the literature by Sriram Vajapeyam^22^ in 2014. Similarly, the entropy can be computed decisionwise for each attribute v which we denote it by H $(S_{v})$.

### Definition: Information gain

An important parameter in machine learning is Information Gain (IG), meant for feature selection in the frame work of decision trees. It evaluates how well a feature classifies the training set of data. The entropy measure provides the extent of impurity present in the dataset. The lower entropy is desirable as it attributes to higher reliability about the dataset. Information gain indicates the significance of a particular feature vector attribute^24^.

2 $$\begin{aligned} IG=H(S)-\sum _{v}{\frac{\left| S_{v}\right| }{ \left| S \right| }} \times H(S_{v}) , \end{aligned}$$

Where |*S*| the total number of instances in dataset, $|S_{v}|$ is the number of instances in dataset for which attribute values v, and H(S) is the entropy of dataset.

#### Steps involved in ID3 algorithm

1. Find the whole dataset’s entropy (using the class distribution).
2. For each feature, determine the entropy categorywise followed by information gain.
3. Prioritize the attributes using information gain. The attributes with higher information gain values are considered to be more important than those with lower values.
4. For any two successive attributes in the priority order say $A_1>A_2$ remove those concepts with extent having attribute $A_2$ but not $A_1$. Repeat this step for all pair of successive attributes in the priority order.
5. Take the union of all the concepts that are retained in the previous step to get the required compressed concepts.

### Definition: Intent weight of a concept

Let the probability of an object $a_{j} (j=1,2,...n)$ to have the attribute $b_{i}, (i=1,2,...m)$ be $P(b_{i}/a_{j})$. For any attribute $b_{i}$, Let $E(b_{i})$ denote its expected value. We use the formulas as in^37,38^ to compute the expected probability of attributes and the weight of intents.

3 $$\begin{aligned}&E(b_{i})= -\sum _{j=1}^{\ n} P(b_{i}/a_{j})log_{3}P(b_{i}/a_{j}), \end{aligned}$$

4 $$\begin{aligned}&w_{i}=\frac{E(b_{i})}{ \sum _{j=1}^ {m } E(b_{i})} , \end{aligned}$$

If (*A*, *B*) denotes a formal concept, then the intent weight namely, weight(B) is given as the mean weights of attributes contained in it.

5 $$\begin{aligned} Weight(B)=\frac{1}{|B|}\sum _{i=1}^{|B|} w_{i} \end{aligned}$$

Here |*B*| denotes the size of the set B. If B has only one attribute, then the intent set is of single attribute else it will be a multi-attribute intent set. We note that the sum of weights $w_{i}$ is 1. We can note that weight(B) does not influence on the deviation between all the intent sets in reality. Due to this fact, the extracted knowledge could not please to the users interests. We use the following approach to get rid of this issue.

### Definition: Deviance analysis

Let (*A*, *B*) be a concept pair. The deviation of attributes weights of an intent B from their average value denoted by *D*(*B*) is known as the deviance measure of the concept (*A*, *B*)^37^ and is defined as below.

6 $$\begin{aligned} D(B)=\left\{ \begin{aligned}&0,&if \ n=1\\&\sqrt{\frac{1}{n-1} \sum _{i=1}^{ n} (w_{i}-weight(B))^2},&else \end{aligned} \right. \end{aligned}$$

### Definition: Percentage error calculation

In^39^, Roocker defined a statistical measure to compute the percentage error of deviance. We use the same to compute the proportion of inaccuracy on deviation measure between the original and reduced concepts. The percentage error calculation formula is:

7 $$\begin{aligned} \delta = \left| \frac{d-d^{\prime }}{v} \right| \times 100, \end{aligned}$$

Where *d* denotes the mean deviance value of intents present in original concepts and $d^{\prime }$ denotes that in reduced concepts and *v* corresponds to the $\max \{d, d^{\prime }\}$. The difference considered here is an absolute one. We consider the model accurate if the absolute error percentage $\delta \le 15 \%$.

## Experiment with ID3 algorithm

We next experiment with the weather data with the ID3 algorithm. As discussed earlier, the ID3 algorithm determines the decision tree structure for any given context by which the priority of attributes can be easily understood in any decision context. Furthermore, ID3 algorithm treats only categorical data types. For this purpose, we modify the data structure presented in Table 7 to suit the required template to treat with ID3 Algorithm. In this regard, the temperature is classified as high, medium and low seasonwise according to the temperature range as specified below in Table 6. These modifications would accordingly form a new many-valued context table for the mean temperature-rain data collected from DSP, Pune, India. The same is presented in Table 7. This context table is then converted into formal context table using categorical attributes and draw the concept lattice.

**Table 6:** Classification of temperature data in seasonwise.

Temperature in °C			
Seasons	Low	Medium	High
Winter	28.9–29.6	29.7–30.3	30.4–31.1
Summer	35–36.5	36.6–38	38.1–39.4
Monsoon	34.1–35.4	35.5–36.7	36.8–38
Autumn	29.5–31.1	31.2–32.7	32.8–34.2

**Table 7:** Many-valued context for temperature-rain data.

Year	Winter	Summer	Monsoon	Autumn	Decision
2008	High	Low	Low	Low	Medium
2009	Low	High	Medium	Low	Low
2010	Medium	High	Low	Medium	High
2011	High	Medium	Low	Medium	Medium
2012	High	High	Medium	Medium	Medium
2013	High	High	Low	Medium	Low
2014	Medium	High	Low	Low	Low
2015	High	Low	Low	Low	Medium
2016	High	High	Low	High	Low
2017	High	High	Low	Low	Medium
2018	High	Medium	Low	Low	Low
2019	Medium	Medium	High	Medium	Medium
2020	Low	Low	Medium	High	High
2021	Medium	Medium	Low	Low	High
2022	Medium	Medium	Low	Low	Medium

**Fig. 3:**
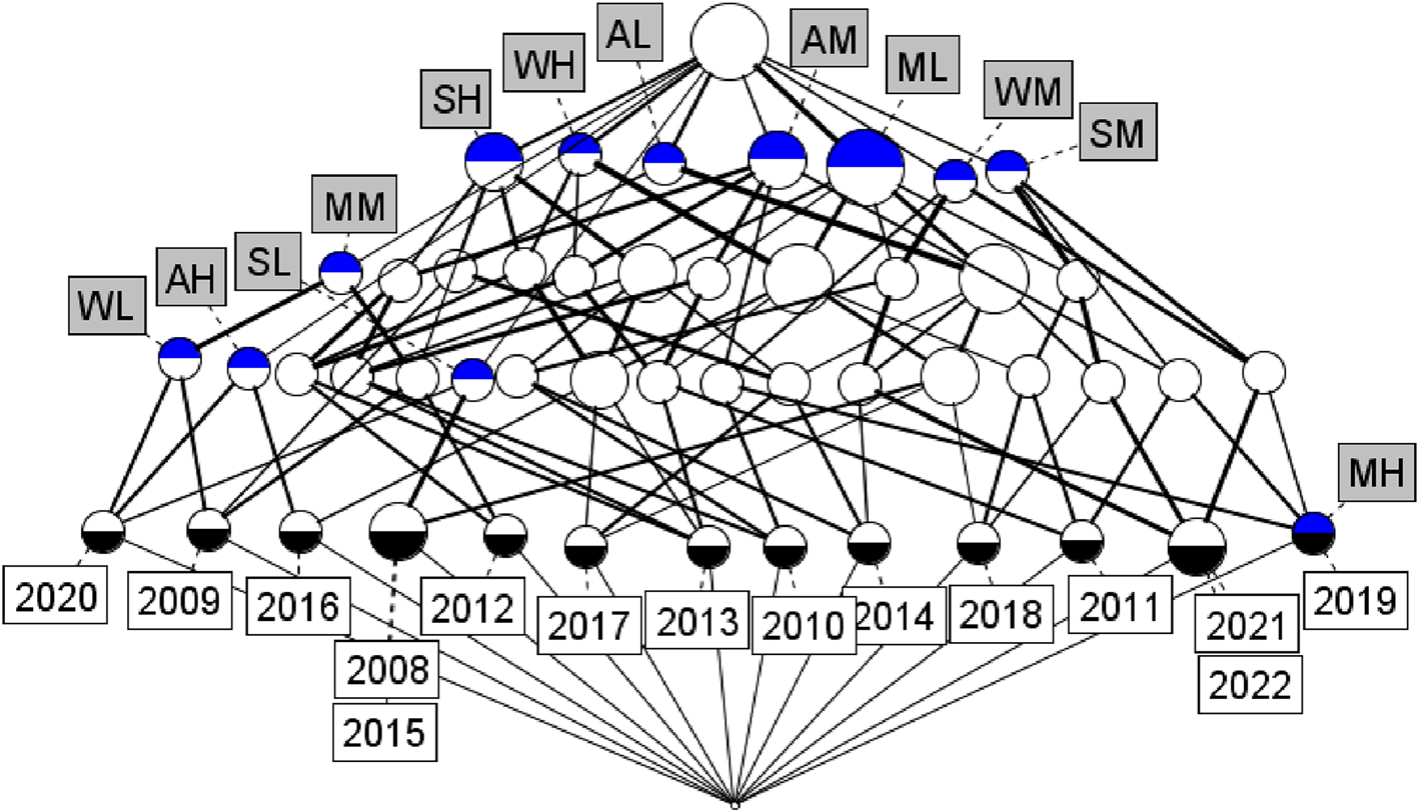
Concept lattice derived from the formal context Table 7.

The formal context table from Table 7 is studied further with the help of FCA to determine its concepts and there by obtain its concept lattice which is presented in Fig. 3.

We can identify from Fig. 3 that, the height of the concept lattice is 5 and contains 50 concepts connected by 114 edges (concepts detailed in Table 8). They are numbered from bottom to top and left to right.

Next we talk about complexity of the proposed method. Let t =number of decisions involve in the datasets. And let v be the number of values present in the multi-valued attributes. Let m, n denote the number of objects and attributes of the datasets respectively. Since the entropy is computed overall objects and each of the multi-valued attributes with respect to all decisions, the complexity of the entropy can be derived as O(nvmt). The sorting of attributes by a exiting sorting algorithms as the computational cost O(nlogn). The compression of concepts can be computed in O(cn)where c denote the number of concepts obtained from the datasets.

## Experimental results

### Results on ID3 values

In this section we compute the ID3 values for the many-valued temperature-rain data presented in Table 7.The readers are advised to refer^18,21,22^ to know about the computation process of entropy values for the attributes. Using Eqs. (1) and (2) from section 4, we get the following results.

Information entropy H(S)=0.9501

IG (Winter): 0.9501–0.7254 = 0.2247

IG (Summer): 0.9501–0.8102 = 0.1399

IG (Monsoon): 0.9501–0.8917 = 0.0584

IG (Autumn): 0.9501–0.8455 = 0.1046

#### ID3 based precedence order of attributes

An attribute that has a greater precision value is ranked highly since it is important in the decision-making context. According to this rule, we get Winter > Summer > Autumn > Monsoon. The precedence order of attribute according to the gain measure is W > S > A > M. The notation W > S means that W is more important than S.

The information gain measure gives the quantifies the amount of information present in a datasets. The higher information gain values attribute to the higher level of useful data present in the datasets. So the priority of seasons W > S > A > M spell out that winter season has higher impact on the rainfall whereas monsoon has the least impact of the rainfall.

The variation of the results to the reality regarding the ranking that Winter > Summer > Autumn > Monsoon, is due to the individual seasons’ contribution to the overall rain yield to the entire year. It’s true that the monsoon seasons have heavy rainfall while compared with the other seasons. But it happens almost uniformly for all the years. So, it has less important role in deciding the high rainfall for the year. At the same time the other seasons play a vital role in making high rainfall for the year. For example, though the summer has few rainfall days, its contribution to the rainfall of the year is significant since it changes the rainfall yield from lower to higher level for the year. The same explanation can be made for the other seasons, too.

**Table 8:** Concepts obtained by using the formal context in Table 9

Concept	({ Extent};{Intent})
C1	( {$\phi$ };{ WL, WM, WH, SL, SM, SH, ML, MM, MH, AL, AM, AH } )
C2	({2019};{MH, AM, WM, SM })
C3	({ 2021, 2022 };{ WM, AL, ML, SM })
C4	( {2011 };{ WH, SM, ML, AM })
C5	( {2018 }; {WH, SM, ML, AL})
C6	( {2014};{WM, SH, ML, AL})
C7	( {2010};{WM, SH, ML, AM})
C8	({2017};{WH, SH, ML, AL})
C9	( {2008, 2015};{WH, SL, ML, AL})
C10	({2013};{WH, SH, ML, AM})
C11	({2012};{WH, SH, MM, AM})
C12	({2016};{WH, SH, ML, AH})
C13	({2009};{WL, SH, MM, AL})
C14	({2020};{WL, SL, MM, AH})
C15	({2019, 2021, 2022};{WM, SM, MH})
C16	({2011, 2019};{SM, MH, AM})
C17	({2018, 2021, 2022};{SM, ML, AL})
C18	({2010, 2019};{WM, MH, AM})
C19	({2014, 2021, 2022};{WM, ML, AL})
C20	({2011, 2018};{WH, SM, ML})
C21	({2010, 2014};{WM, SH, ML})
C22	({2008, 2015, 2017, 2018};{WH, ML, AL})
C23	({2014, 2017};{SH, ML, AL})
C24	({2011, 2013};{WH, ML, AM})
C25	({2010, 2013};{SH, ML, AM})
C26	({2013, 2016, 2017};{WH, SH, ML})
C27	({2012, 2013};{WH, SH, AM})
C28	({2008, 2015, 2020};{SL})
C29	({2009, 2012};{SH, MM})
C30	({2016, 2020};{AH})
C31	({2009, 2020};{WL, MM})
C32	({2011, 2018, 2021, 2022};{SM, ML})
C33	({2010, 2014, 2021, 2022};{WM, ML})
C34	({2008, 2014, 2015, 2017, 2018, 2021, 2022};{ML, AL})
C35	({2010, 2011, 2013};{ML, AM})
C36	({2008, 2011, 2013, 2016, 2015, 2016, 2018};{WH, ML})
C37	({2010, 2013, 2014, 2016, 2017};{SH, ML})
C38	({2011, 2012, 2013};{WH, AM})
C39	({2010, 2012, 2013};{SH, AM})
C40	({2009, 2014, 2017};{SH, AL})
C41	({2012, 2013, 2016, 2017};{WH, SH})
C42	({2009, 2012, 2020};{WL, MM})
C43	({2011, 2018, 2019, 2021, 2022};{SM, MH})
C44	({2010, 2014, 2019, 2021, 2022};{WM, MH})
C45	({2008, 2010, 2011, 2013, 2014, 2015, 2016, 2017, 2018, 2021, 2022};{ML})
C46	({2010, 2011, 2012, 2013, 2019};{MH, AM})
C47	({2008, 2009, 2014, 2015, 2017, 2018, 2021, 2022};{AL})
C48	({2008, 2011, 2012, 2013, 2015, 2016, 2017, 2018};{WH})
C49	({2009, 2010, 2012, 2013, 2014, 2016, 2017};{SH})
C50	({2008, 2009, 2010, 2011, 2012, 2013, 2014, 2015, 2016, 2017, 2018, 2019, 2020, 2021, 2022};$\{ \phi \}$)

The set of discarded precedence relation order is then listed. According to the precedence relation, A>M implies that attribute A is the direct predecessor of attribute M.

#### Precedence relation-based discarded concepts

A>M

C20, C21, C26, C27, C28, C29, C30, C31, C32, C33, C36, C37, C38, C39, C40, C41, C42, C43, C44, C45, C46, C47, C48, C49.

S>A

C15, C18, C19, C20, C21, C22, C24, C26, C28, C29, C30, C31, C32, C33, C34, C35, C36, C37, C38, C41, C42, C43, C44, C45, C46, C47, C48, C49.

W>S

C16, C17, C18, C19, C22, C23, C24, C25, C28, C29, C30, C31, C32, C33, C34, C35, C36, C37, C38, C39, C40, C42, C43, C44, C45, C46, C47, C48, C49.

The following is the compressed concepts for each pairwise precedence relation.

#### Concept retained after precedence relation

A>M

C1, C2, C3, C4, C5, C6, C7, C8, C9, C10, C11, C12, C13, C14, C15, C16, C17, C18, C19, C22, C23, C24, C25, C34, C35, C46.

S>A

C1, C2, C3, C4, C5, C6, C7, C8, C9, C10, C11, C12, C13, C14, C16, C17, C23, C25, C27, C39, C40.

W>S

C1, C2, C3, C4, C5, C6, C7, C8, C9, C10, C11, C12, C13, C14, C15, C20, C21, C26, C27, C41.

The final reduced collection of concepts includes only those concepts that obey to the precedence order relation with regard to at least one precedence order pair of characteristics. Concepts are eliminated from the set if they do not follow the precedence order relation of attributes with regard to any of the precedence order pairs.

#### 33 concepts have been reduced, as follows

C1, C2, C3, C4, C5, C6, C7, C8, C9, C10, C11, C12, C13, C14, C15, C16, C17, C18, C19, C20, C21, C22, C23, C24, C25, C26, C27, C34, C35, C39, C40, C41, C46.

In any given situation, we expect that an object’s attributes should adhere to at least one precedence order pair relation. Therefore, this must also be true for every concept’s attribute or intent. The union of these concepts resulting from each precedence order pair must then be the intended set of final compressed concepts. Thus out of the 50 total number of original concepts, after reduction the number of concepts obtained is 33.

#### Measurement of quality of compressed concepts

In this section, determine each attribute’s weight, probability, and expectation using Eqs. (3) and (4) we get the below Table 9.

**Table 9:** Acquisition method for the single intent weight value.

	*P*(*X*)	*E*(*X*)	$W_{i}$
WL	0.133	0.245	0.072
WM	0.333	0.333	0.099
WH	0.533	0.305	0.09
SL	0.2	0.293	0.087
SM	0.333	0.333	0.099
SH	0.467	0.324	0.096
ML	0.733	0.207	0.061
MM	0.2	0.293	0.087
MH	0.067	0.164	0.049
AL	0.533	0.305	0.090
AM	0.333	0.333	0.099
AH	0.133	0.245	0.072

Next, we use Eqs. (5) and (6) to calculate the relevant of concepts, which is the Intent Average Value (IAV), weight, and deviation for each concept. The outcomes are shown in Table 10 .

**Table 10:** The weight values of intents and their deviation values for the original concepts.

Concepts	Intent	IAV	Weight	D(I)
C1	WL, WM, WH, SL, SM, SH, ML, MM, MH, AL, AM, AH	0.083	0.083	0.015
C2	MH, AM, WM, SM	0.086	0.086	0.017
C3	WM, AL, ML, SM	0.065	0.065	0.017
C4	WH, SM, ML, AM	0.087	0.087	0.017
C5	WH, SM, ML, AL	0.085	0.085	0.016
C6	WM, SH, ML, AL	0.087	0.087	0.017
C7	WM, SH, ML, AM	0.087	0.087	0.017
C8	WH, SH, ML, AL	0.084	0.084	0.014
C9	WH, SL, ML, AL	0.072	0.072	0.017
C10	WH, SH, ML, AM	0.087	0.087	0.017
C11	WH, SH, MM, AM	0.093	0.093	0
C12	WH, SH, ML, AH	0.08	0.08	0.017
C13	WL, SH, MM, AL	0.084	0.084	0.01
C14	WL, SL, MM, AH	0.086	0.086	0.013
C15	WM, SM, MH	0.082	0.082	0.029
C16	SM, MH, AM	0.082	0.082	0.029
C17	SM, ML, AL	0.083	0.083	0.02
C18	WM, MH, AM	0.082	0.082	0.029
C19	WM, ML, AL	0.083	0.083	0.02
C20	WH, SM, ML	0.083	0.083	0.02
C21	WM, SH, ML	0.085	0.085	0.021
C22	WH, ML, AL	0.081	0.081	0.054
C23	SH, ML, AL	0.082	0.082	0.17
C24	WH, ML, AM	0.083	0.083	0.02
C25	SH, ML, AM	0.085	0.085	0.021
C26	WH, SH, ML	0.082	0.082	0.017
C27	WH, SH, AM	0.095	0.095	0
C28	SL	0.087	0.087	0
C29	SH, MM	0.091	0.091	0
C30	AH	0.072	0.072	0
C31	WL, MM	0.08	0.08	0.01
C32	SM, ML	0.08	0.08	0.028
C33	WM, ML	0.08	0.08	0.028
C34	ML, AL	0.076	0.076	0.02
C35	ML, AM	0.08	0.08	0.028
C36	WH, ML	0.076	0.076	0.02
C37	SH, ML	0.079	0.079	0.026
C38	WH, AM	0.094	0.094	0
C39	SH, AM	0.097	0.097	0
C40	SH, AL	0.093	0.093	0
C41	WH, SH	0.093	0.093	0
C42	WL, MM	0.08	0.08	0.010
C43	SM, MH	0.074	0.074	0.035
C44	WM, MH	0.074	0.074	0.035
C45	ML	0.061	0.061	0
C46	MH, AM	0.074	0.074	0.035
C47	AL	0.09	0.09	0
C48	WH	0.09	0.09	0
C49	SH	0.096	0.096	0
C50	$\phi$	0	0	0

In Table 11, we tabulate the calculation related to reduced concepts.

**Table 11:** The retained concepts of importance deviation value and intent weight value.

Concepts	Intent	IAV	Weight	D$(I^{\prime })$
C1	WL, WM, WH, SL, SM, SH, ML, MM, MH, AL, AM, AH	0.083	0.083	0.015
C2	MH, AM, WM, SM	0.086	0.086	0.017
C3	WM, AL, ML, SM	0.065	0.065	0.017
C4	WH, SM, ML, AM	0.087	0.087	0.017
C5	WH, SM, ML, AL	0.085	0.085	0.016
C6	WM, SH, ML, AL	0.087	0.087	0.017
C7	WM, SH, ML, AM	0.087	0.087	0.017
C8	WH, SH, ML, AL	0.084	0.084	0.014
C9	WH, SL, ML, AL	0.072	0.072	0.017
C10	WH, SH, ML, AM	0.087	0.087	0.017
C11	WH, SH, MM, AM	0.093	0.093	0
C12	WH, SH, ML, AH	0.08	0.08	0.017
C13	WL, SH, MM, AL	0.084	0.084	0.01
C14	WL, SL, MM, AH	0.086	0.086	0.013
C15	WM, SM, MH	0.082	0.082	0.029
C16	SM, MH, AM	0.082	0.082	0.029
C17	SM, ML, AL	0.083	0.083	0.02
C18	WM, MH, AM	0.082	0.082	0.029
C19	WM, ML, AL	0.083	0.083	0.02
C20	WH, SM, ML	0.083	0.083	0.02
C21	WM, SH, ML	0.085	0.085	0.021
C22	WH, ML, AL	0.081	0.081	0.054
C23	SH, ML, AL	0.082	0.082	0.17
C24	WH, ML, AM	0.083	0.083	0.02
C25	SH, ML, AM	0.085	0.085	0.021
C26	WH, SH, ML	0.082	0.082	0.017
C27	WH, SH, AM	0.095	0.095	0
C34	ML, AL	0.076	0.076	0.02
C35	ML, AM	0.08	0.08	0.028
C39	SH, AM	0.097	0.097	0
C40	SH, AL	0.093	0.093	0
C41	WH, SH	0.093	0.093	0
C46	MH, AM	0.074	0.074	0.035

### Validation process-calculation of percentage error

The results are validated with the help of error measure. The error rate represents the percentage error of deviance for the compressed concepts with respect to the original ones for he temperature-rain data.

$\sum D(I) = 0.7760$, $d=0.7760/50 = 0.0155$ and $\sum D(I^{\prime }) = 0.5840$, $d^{\prime } = 0.5840/33 = 0.0177$ Where *I*, $I^{\prime }$ stands for the intents of original and compressed concepts respectively.

Here *v* = $$max\{d,d^{\prime }\}$$ = $$max\{0.0155, 0.0177\}$$ = 0.0177.

Using these values, in equation(7), we get

$$\delta$$ = $$\left| \frac{d-d^{\prime }}{v}\right| \times 100$$

$$= 0.1248 \approx 12\%$$

It is observed that there is a negligible percentage error $\delta =12\%$ of weight deviance between compressed concepts. Since the percentage of error $\delta <15\%$, the threshold level for better model which we discussed earlier, we can conclude that the removed concepts have higher deviations by means of their intent weights than the existing (compressed) concepts.Consequently, we can draw the conclusion that the compressed concepts can be regarded as more relevant.

FCA can be an effective technique for investigating and displaying correlations particularly with smaller, more precisely defined datasets with distinct categories. However, when the dataset is huge, the representation of data with the use of FCA becomes more complex and difficult to understand. To overcome such limitations, preprocessing (such as discretisation) and supplementary techniques may be needed when applying it to continuous, sizable, or noisy datasets. This preprocessing stage is adopted by the authors in this article also. Similarly, ID3 algorithm also has limitations with regard to scalability, handling continuous data, and preventing overfitting, even though it can be helpful in some situations, particularly with smaller, simpler datasets and classification problems. Such issues are resolved here in the preprocessing stages.

Climate data is not uniform throughout any region or country. So the accuracy of data lacks as the region is expanded. Se we have collected data from a particular place in order to understand an analysis the data more accurately. If needed the analysis can be carryout for any specific region.

We have considered 15 years of temperature and rain data. Table 11 contains the important concepts that are retained. In our analysis, we find that the attribute ML (Monsoon Low temperature) occurs 21 times and it is mostly available in the 15 years period which implies that it is a vital attribute in the rainfall prediction. Next important attribute is SH (Summer High temperature) which is found 16 times. In a similar way we can understand the importance of remaining attributes. We believe that the analysis of the reduced concepts can help the farmers and other service providers who need the rainfall prediction data in understanding climatic changes that happen before rainfall.

## Conclusions

The novelty of this article lies in the usage of ID3 algorithm through FCA approach to understand the temperature-rain data. Such an approach is new in the prediction models. In this article we have studied the temperature-rain data seasonwise and overall (yearwise). This paper presents the application of FCA in mining the temperature-rain data from the district of Vellore, Tamil Nadu, India. The dataset contained originally about 145 concepts. After preprocessing the context there were about 50 concepts. The usage of the ID3 algorithm further reduced it to 33 concepts. So we can infer more meaningful information with less number of concepts easily.

The key contributions of this article are as follows.

- Three way decision context is dealt effectively with the use of ID3 algorithm.
- Priorities of seasons are obtained with respect to the decision ’rainfall’.
- The number of concepts are reduced using the priority of attributes.
- The reduced concepts are validated with the use of intent weight parameter which correspond to the quality of reduced concepts.

As a future work, this model can be apply to the datasets involving decisions and with attribute values changing continuously. For example, in the field of medical diagnosis, to predict the sickness, this model can be apply. As another example, you can use this model to predict lower and higher economics in the country using attributes related to the economy, which are mostly continuous and natural.

## Data Availability

Weather data collected from Indian Meteorological Department, National Data Center (NDC), Pune (The Data Supply Portal http://dsp.imdpune.gov.in/).
